# Impact of Obstructive Sleep Apnea on Cardiovascular Health: A Systematic Review

**DOI:** 10.7759/cureus.71940

**Published:** 2024-10-20

**Authors:** Swati Thareja, Ramya Mandapalli, Fahmida Shaik, Arushee Rajeev Pillai, Gowrishankar Palaniswamy, Sweta Sahu, Sri Pranita Cherukuri, Salma Younas

**Affiliations:** 1 Internal Medicine, The Hans Foundation, Delhi, IND; 2 Internal Medicine, Apollo Institute of Medical Sciences and Research, Hyderabad, IND; 3 Internal Medicine, Gandhi Medical College, Secunderabad, Hyderabad, IND; 4 Internal Medicine, MS Ramaiah Medical College, Bangalore, IND; 5 Medicine, Saveetha Medical College and Hospital, Chennai, IND; 6 Internal Medicine, JJM Medical College, Davanagere, IND; 7 Mailman School of Public Health, Columbia University, New York, USA; 8 Pharmacy, Punjab University College of Pharmacy, Lahore, PAK

**Keywords:** cardiovascular health, cardiovascular risks, comorbidities, coronary artery disease, hypertension, sleep apnea, sleep disorder

## Abstract

Obstructive sleep apnea (OSA) is characterized by recurrent interruptions in breathing during sleep and is associated with a wide range of significant health concerns, particularly cardiovascular complications. This review focuses on the two primary forms of sleep apnea, namely, obstructive and central, with OSA being the most prevalent and commonly linked to conditions, such as hypertension, arrhythmias, and heart failure. Intermittent hypoxemia, activation of the sympathetic nervous system, systemic inflammation, and remodeling of the ventricles are some of the pathophysiological mechanisms that link OSA to cardiovascular disease (CVD). These all lead to higher oxidative stress and endothelial dysfunction. These processes elevate the risk of CVDs, including hypertension, coronary artery disease, and stroke. This systematic review followed the Preferred Reporting Items for Systematic Reviews and Meta-Analyses (PRISMA) guidelines and examined studies published over the last 15 years. The search strategy utilized key terms such as “sleep disorder,” “apnea,” “hypertension,” “cardiovascular disease (CVD),” and “treatment outcomes” across multiple databases, including PubMed, Scopus, Embase, and the Cochrane Library. A total of 128 unique studies were identified, of which 10 met the inclusion criteria and were included in the final analysis. These studies consistently highlighted the significant role OSA plays in exacerbating cardiovascular risks, particularly in patients with comorbid conditions such as obesity, hypertension, and diabetes.

## Introduction and background

Sleep apnea is a common sleep disorder, characterized by repeated episodes of complete cessation of respiration during sleep. This condition is associated with rather serious health risks, with a focus on cardiovascular health, in particular. In terms of etiology, sleep apnea can be classified into two types: obstructive sleep apnea (OSA) and central sleep apnea (CSA). OSA, the more common form of sleep apnea, is caused by the blockage of the upper airway and is linked to systemic health issues, including hypertension, arrhythmias, metabolic disorders, and cardiovascular diseases (CVDs) [1,2]. CSA is the second form of sleep apnea, which is considered a less common disease, caused by haphazard impulses of the brain to the muscles that control the respiratory system. It is essential to mention that chronic intermittent hypoxia and sleep fragmentation caused by OSA are widely recognized as the major pathophysiological pathways linking sleep apnea to the body response leading to severe cardiovascular health problems [1]. Given the role of sleep apnea in disrupting normal breathing and oxygen flow, it has been recognized as a major contributor to CVDs. This link is largely attributed to the physiological stress caused by repeated episodes of hypoxia, leading to increased blood pressure, systemic inflammation, and oxidative stress, all of which contribute to the progression of cardiovascular conditions [1].

CVD is still a leading cause of morbidity and mortality worldwide, and sleep apnea is a potentially modifiable risk factor for developing heart problems that has been drawing more growing attention over the past decades [3]. In addition, there is more and more evidence to believe that untreated sleep apnea can make pre-existing cardiovascular problems even worse and, to some extent, contribute to the development of new CVDs [2]. The health conditions may be caused by a number of factors, the findings of the recent studies have emphasized the roles of several factors, such as increased oxidative stress, systemic inflammation, as well as sympathetic neural activation, stressing the inexplicit etiology of the latter factors in stimulating various adverse CVDs [4].

Despite its prevalence, reported in 13% of men and 6% of women, OSA is likely underdiagnosed, particularly in patients with mild symptoms, contributing to its delayed recognition as a cardiovascular risk factor. This underdiagnosis complicates the relationship between OSA and CVD. The prevalence of OSA increases with age, obesity, and comorbidities that independently contribute to cardiovascular risk. On the other hand, CSA is less common in people but can occur in those with heart failure and other heart diseases. These conditions complicate the relationship of sleep-disordered breathing to cardiovascular health [5].

Research on sleep apnea in association with CVD has been extensive across groups. There are many studies about OSA suggested the risk of heart attacks, coronary heart disease, heart failure, irregular seizures, stroke, and even sudden cardiac death related to the disease. Notably, OSA is simultaneously the cause of increased risks of getting associated heart diseases and the risk factor that worsens the chances for patients with a previous heart disease. Due to the direct correlation of the severities of obesity and OSA, the diseases are increasingly anticipated by the year to come [6].

Pathophysiology

The involvement of sleep apnea and apnea-hypopnea in the increased risk of CVD is complicated and multifactorial and, although poorly understood, involves several related mechanisms of interaction. The basics of most of these mechanisms include cyclical hypoxemia, hypercapnia, sleep arousal in apnea, the effects of oxidative stress caused by the frequency of these states, systemic inflammation, and activation of the sympathetic nervous system [7].

The outstanding feature of OSA is intermittent hypoxia, which occurs when apneic events are followed by reoxygenation. Such a continuous exposition to intermittent hypoxia results in the upregulation of oxidative stress, during which reactive oxygen species are overproduced, outnumbering the amount of antioxidants in the body [8]. The mechanism is known to damage the endothelial cells, impairing vascular function, and maintaining the dynamic increase in atherosclerosis, a significant cause of the emergence of CVDs. In addition, hypoxia-reoxygenation cycles lead to the activation of hypoxia-inducible factors (HIFs), which are involved in the triggering of inflammatory pathways and intensification of endothelial dysfunction [9].

The activity of the sympathetic nervous system rises due to recurrent episodes of hypoxia and occasional occurrence of hypercapnia in OSA patients. The increased sympathetic tone not only stays present with concomitant wakefulness, increased blood pressure also known as hypertension, higher heart rate, and cardiac remodeling [10,11]. Moreover, continuous activation of the sympathetic chain leads to vasoconstriction, raised afterload, and heightened myocardial oxygen demand, all increasing the risk of IHD, heart failure, and arrhythmias, which results in deterioration of the already existing condition of cardiac patients, such as vital worsening of hypertension or heart failure patients [12].

OSA is known to be associated with continuous low-grade inflammation, as repetitive episodes of hypoxia and sleep disturbance occur. A higher level of inflammatory cytokines have been recorded in patients with OSA: tumor necrosis factor-alpha, interleukin-6, and C-reactive protein. Inflammation plays a key role in the etiology of CVD, promoting atherosclerosis, the instability of plaques, and thrombosis formation. Moreover, frequent hypoxia coupled with sleep disruption results in endothelial dysfunction, which prevents correct vasodilation and increases the risk of hypertension or cardiovascular events [13].

Sleep apnea leads to hemodynamic changes due to repeated episodes of negative intrathoracic pressure during obstructive events, which may result in the cardiac remodeling of the heart. OSA is associated with left ventricular hypertrophy, diastolic dysfunction, and left atrium dilatation. However, these changes lead to an increase in the possibility of heart failure and onset of atrial fibrillation. Furthermore, autonomic arousal and hypoxemia in OSA promote the development of arrhythmias, such as atrial fibrillation, ventricular tachycardia, and bradyarrhytmias. However, in CSA, which is common in patients with heart failure, there is an increased likelihood of nocturnal arrhythmias and sudden death from cardiac causes [14].

Contributing factor for particular CVD

OSA is an independent risk factor for hypertension, as the severity of the condition is directly proportional to the increase in blood pressure. Thus, nocturnal sympathetic overactivity and hypoxemia caused by sleep apnea result in sustained high nighttime blood pressure, which may promote the development of hypertension not only during the daytime but also at night. In this way, OSA is one of the etiologies of resistant hypertension, which is characterized by the failure to achieve blood pressure control despite taking three more antihypertensive medications [15,16].

Intermittent hypoxia and systemic inflammation associated with OSA stimulate the development of atherosclerosis. That is why patients with OSA are more likely to develop myocardial infarction and have a worse prognosis after acute coronary syndrome. The biologically plausible mechanisms for the connection between OSA and CAD are increased oxidative stress, endothelial dysfunction, and heightened platelet reactivity [17].

**Figure 1:**
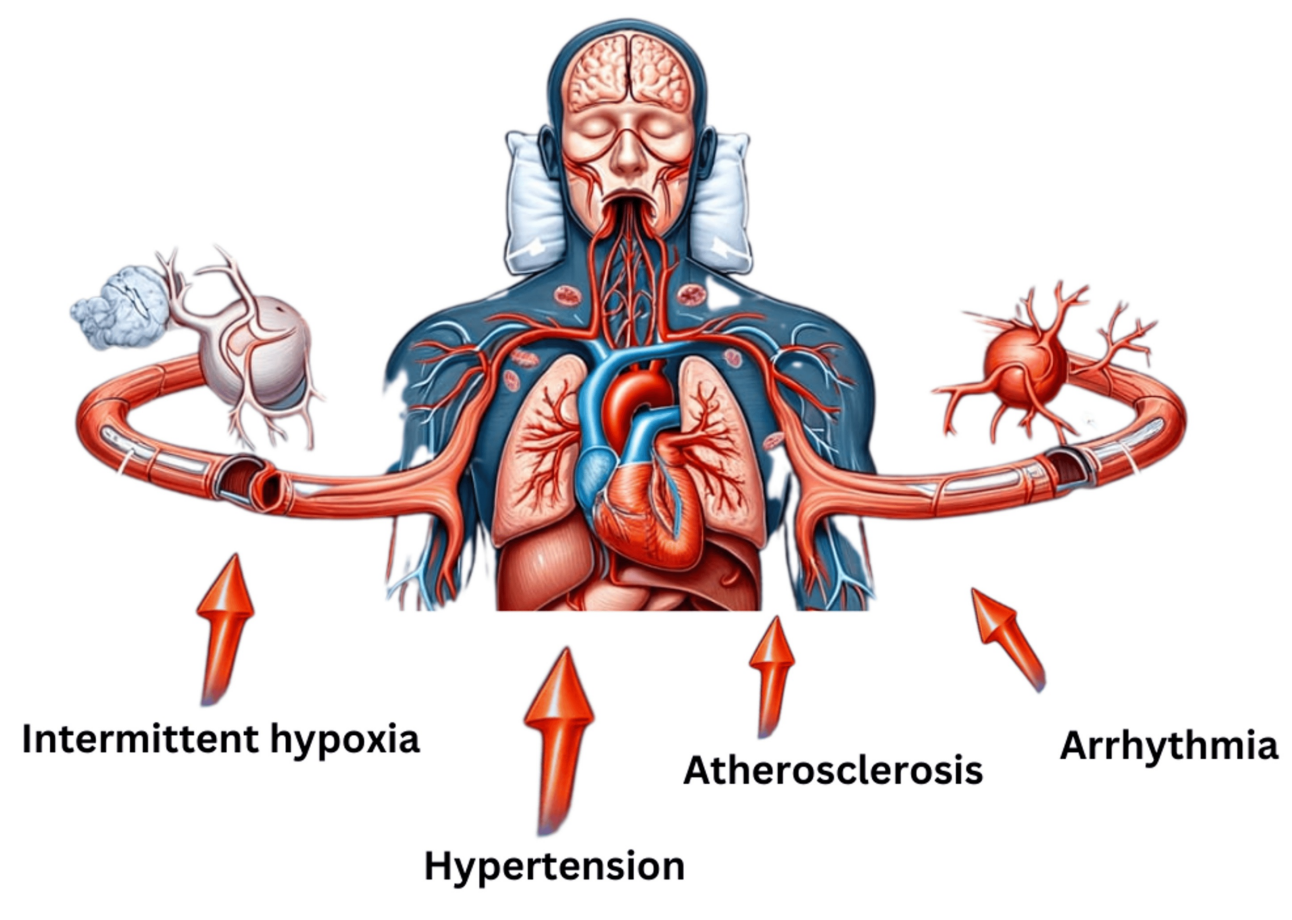
Effect of sleep apnea on cardiovascular health Image credits: Swati Thareja, Sweta Sahu, Salma Younas The authors used the DALL·E AI tool to generate the initial version of Figure 1 based on a specific prompt. Subsequent edits were made by the authors to align the figure with the requirements.

OSA and CSA are frequent in patients having heart failure and they accelerate the course of the disease. The repetitive episodes of hypoxemia heightened sympathetic activity, and negative intrathoracic pressure caused by sleep apnea is a considerable burden for the faltering heart, which leads to more rapid worsening of the cardiac condition and increased level of hospitalization. CSA is especially alarming in patients with heart failure as it is associated with Cheyne-Stokes respiration, which is known for its poor prognosis and increased mortality rates [18] (Figure 1).

There is a connection between sleep apnea and atrial fibrillation, and OSA is considered one of the major risk factors for both the occurrence and relapses of atrial fibrillation. The processes that participate in this connection include malfunctioning of the autonomic nervous system, atrial remodeling, and oxidative harm from hypoxemia. In addition, sleep apnea is one of the factors that increase the risk of ischemic cerebrovascular accident, both independently and through its connection with atrial fibrillation and systemic hypertension [19]. The aim is to analyze the effects of sleep apnea on cardiovascular health, with a focus on understanding the mechanisms driving these relationships and highlighting the importance of early diagnosis and intervention.

## Review

Methodology

**Figure 2:**
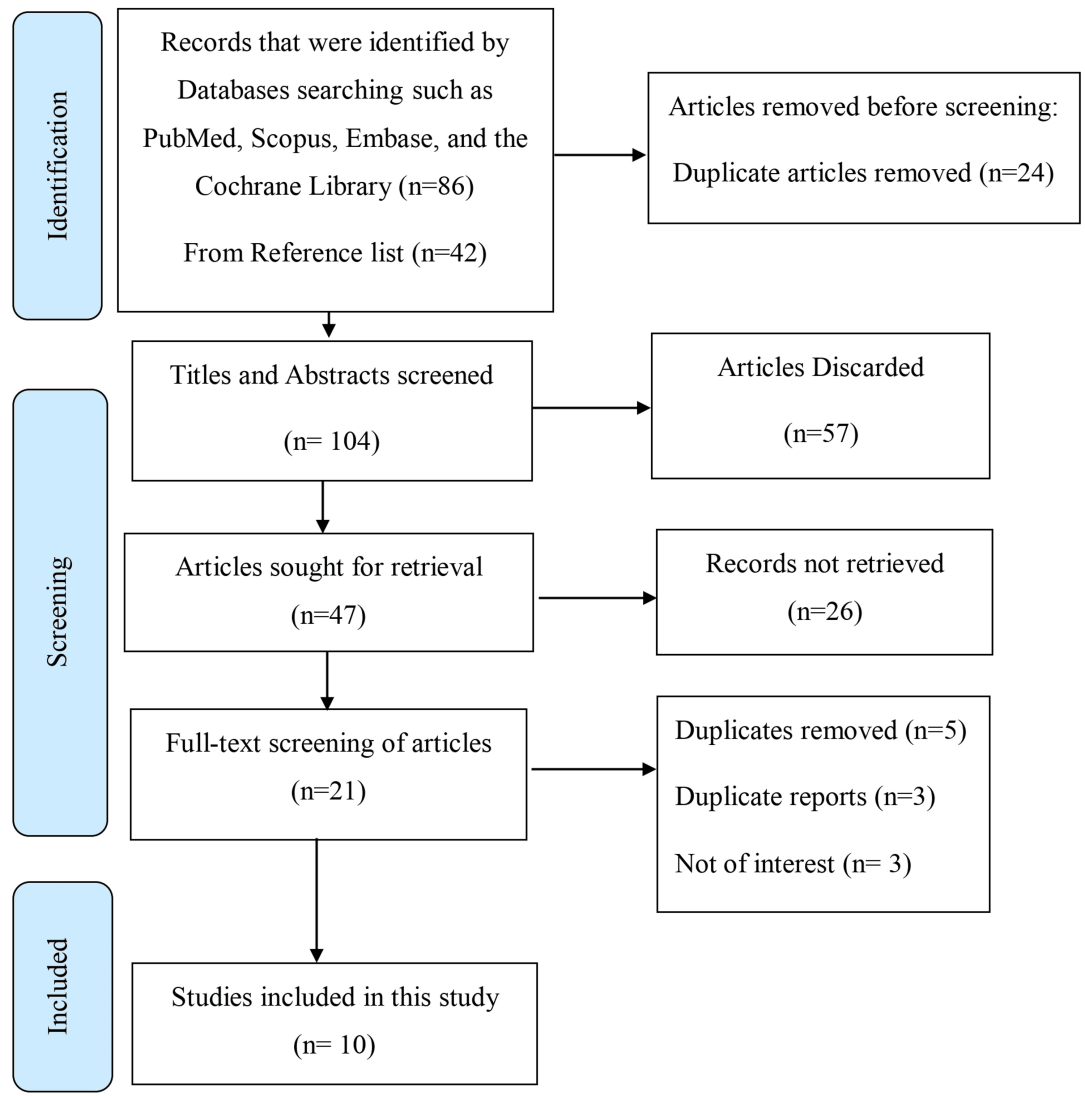
PRISMA flow diagram PRISMA: Preferred Reporting Items for Systematic Reviews and Meta-Analyses

The current methodology is based on a systematic review approach, which allows for identifying whether a research problem is underinvestigated while following the specific rules of the Preferred Reporting Items for Systematic Reviews and Meta-Analyses (PRISMA). A PRISMA flow diagram is used to describe the process of selecting studies visually (Figure 2).

Inclusion and Exclusion Criteria

Studies specifically evaluating the relationship between sleep apnea (both OSA and CSA) and cardiovascular health outcomes were included. Observational, experimental, and longitudinal studies were considered if they provided sufficient data on cardiovascular outcomes in individuals diagnosed with sleep apnea. Only studies published in English within the past 15 years were included.

The studies focusing on the cardiovascular effects of sleep apnea, published in the last 15 years, and written in English, were included. Observational, experimental, and longitudinal studies that used proper diagnostic tools, such as polysomnography, were eligible for inclusion.

Systematic reviews, meta-analyses, and experimental research were excluded, as well as studies with insufficient cardiovascular data. Articles focusing solely on pediatric populations, conference abstracts, and non-peer-reviewed publications were also excluded to ensure the quality and relevance of the data.

Search Strategy

The search strategy was designed to make sure that all relevant research on the effects of sleep apnea on cardiovascular health would be included. For this purpose, several databases were used, including PubMed, Scopus, Embase, and the Cochrane Library. The systematic combination of several keywords and Medical Subject Headings (MeSH) related to sleep apnea, CVD, hypertension, heart failure, cardiovascular arrhythmias, and stroke was used (Table 1). The search strategy included a manual examination of the reference list of relevant reviews, to potentially include the papers that were not found in the systematic search of databases.

**Table 1:** Keywords used for search strategy CVD: cardiovascular disease, CAD: coronary artery disease

Category	Keywords and MeSH terms
Sleep apnea	" leep disorder," " sleep issues," "apnea"
Hypertension	"pulmonary hypertension," "secondary hypertension," "high blood pressure"
Cardiovascular health	"CVD," "CAD," "high blood pressure”
Treatment response	"therapeutic response," " efficacy treatment," "treatment outcomes," "effectivity"

Data Management

Data management techniques were used to ensure the accuracy and reproducibility of the results of the study. For this purpose, all data collected from the included studies were verified by other reviewers to minimize the risk of errors. For the secure storage of data, a database protected by encryption was used, and access to it was only available to the research team. 

Results

The systematic review assessed the impact of sleep apnea on cardiovascular health by examining both diagnostic and therapeutic outcomes. Ten studies that met the inclusion criteria were included in the final review (Table 2). The primary aim was to evaluate how sleep apnea influences cardiovascular risk factors, including hypertension, heart disease, and stroke, as well as treatment outcomes. Across the studies, it was consistently found that sleep apnea significantly affects cardiovascular risks and patient responses to treatment.

**Table 2:** Characteristics of the study included OSA: obstructive sleep apnea, OSAHS: obstructive sleep apnea-hypopnea syndrome, BMI: body mass index, BP: blood pressure, CVP: central venous pressure, CVD: cardiovascular disease, MI: myocardial infarction, OR: odds ratio, CI: confidence interval, AHI: apnea-hypopnea index, REM: rapid eye movement, NREM: non-rapid eye movement, BZRAs: benzodiazepine receptor agonists

Reference	Country	Study design	Sample size	Comorbidities	Results	Key findings
Wilson et al., 2018 [6]	Australia	Cross-sectional study	80	Gestational diabetes, pre-eclampsia	The prevalence of sleep-disordered breathing was 1.4 times higher in the cases compared to controls.	No significant correlation was found between prenatal pre-eclampsia/hypertension and sleep-disordered breathing. However, women with prenatal hypertension/pre-eclampsia had more than double the prevalence of sleep-disordered breathing.
Saraei et al., 2020 [19]	Iran	Cross-sectional study	281	Obesity, hypertension	BMI was 26.9 ± 3.9 kg/m²; 59.9% had a combination of risk factors like arterial BP ≥140/90 mmHg, BMI > 35 kg/m², and age > 50 years.	High BMI and multiple risk factors are significantly associated with sleep apnea in hypertensive and obese populations.
Poka-Mayap et al., 2020 [20]	Cameroon	Cross-sectional study	359	Obesity, stroke, heart failure, epilepsy, hypertension	The prevalence of high-risk OSA was 64.1%, with higher rates in males (78.3%) than females (53%). Moderate-to-severe OSA was significantly associated with hypertension.	Sleep apnea, particularly moderate-to-severe cases, is strongly associated with hypertension, particularly in males.
Kolluri et al., 2020 [21]	USA	Retrospective study	264	Diabetes mellitus, pulmonary embolism	22.7% of patients had high CVP, 26.9% diagnosed with OSA. High CVP was associated with older age and diabetes mellitus.	No significant difference in OSA prevalence was found. Age and diabetes mellitus are strong predictors of high CVP.
Karhu et al., 2021 [22]	Finland	Longitudinal study	2355	Hypertension, diabetes mellitus, CVD	Elevation in oxygen desaturation index and desaturation duration was more pronounced in individuals with cardiovascular comorbidities.	Individuals with pre-existing diabetes or CVD face an increased risk of worsening intermittent hypoxemia, contributing to poorer cardiovascular outcomes.
Kaya et al., 2020 [23]	Turkey	Cross-sectional study	266	Hypertension	Hypertension was associated with OSA, with risk factors including advanced age, increased Epworth Sleepiness Scale, and higher oxygen desaturation index.	OSA is significantly associated with hypertension, with factors like age and oxygen desaturation being critical in identifying at-risk individuals.
Coussa-Koniski et al., 2020 [24]	Lebanon	Longitudinal study	663	Hypertension, diabetes mellitus, CVD, obesity, MI	90% of OSA patients had comorbidities, with 60% having 2-4 comorbidities. Older age, male sex, and obesity were linked to hypertension and diabetes.	Men had higher OSA severity, while comorbidities were more common in women. Snoring and sleep apnea were independently associated with severe OSA.
Barreto et al., 2020 [25]	Brazil	Cohort study	102	Physical inactivity, hypertension, diabetes mellitus, CVD	One-third of cases involved a wake-up stroke; 50% had moderate-to-severe OSA. Type 2 diabetes was independently associated with stroke (OR = 2.76; CI 1.10–6.05).	Type 2 diabetes is a strong predictor of stroke in individuals with sleep apnea. Moderate-to-severe OSA significantly increases stroke risk.
Almeneessier et al., 2020 [26]	Saudi Arabia	Cross-sectional study	32	Hypertension	The average AHI was 40.1 ± 27.6 episodes per hour. No significant difference in desaturation or respiratory events between REM and NREM sleep.	A high AHI was observed, and the oxygen desaturation index predicted changes in systolic BP, highlighting the need for managing desaturation levels in OSA patients.
Hein et al., 2019 [27]	Belgium	Cross-sectional study	1272	Hypertension	Short sleep duration (<5 hours) significantly increased the risk of hypertension. A sleep fragmentation index >18 per hour also raised the likelihood of hypertension. Prolonged use of short or intermediate half-life BZRAs was linked to higher hypertension incidence in patients with sleep disorders.	Hypertension was present in 30.03% of individuals with sleep problems. Key risk factors included short sleep duration (<5 hours), decreased sleep efficiency (<65%), increased sleep fragmentation (index >18 per hour), and the use of short or intermediate half-life BZRAs.

Risk-of-Bias Assessment

Risk of bias was assessed using Cochrane RoB 2 and ROBINS-I tools. It is based on the types of studies included and potential biases. The table considers factors such as study design, sample size, blinding, selection criteria, and potential sources of bias like funding or conflict of interest. Each study is assessed for key risk factors that could impact the validity of the findings (Table 3).

**Table 3:** Risk-of-bias assessment using the Cochrane RoB 2 and ROBINS-I tools Cochrane RoB 2: Cochrane Risk of Bias 2, ROBINS-I: Risk of Bias in Non-randomized Studies of Interventions

Study reference	Study design	Sample size	Selection bias	Detection bias	Confounding	Attrition bias	Reporting bias	Overall risk
Wilson et al., 2018 [6]	Cross-sectional	80	Low	High	Moderate	Low	Moderate	Moderate
Saraei et al., 2020 [19]	Cross-sectional	281	Low	High	Moderate	Low	Low	Moderate
Poka-Mayap et al., 2020 [20]	Cross-sectional	359	Low	High	High	Low	Low	High
Kolluri et al., 2020 [21]	Retrospective	264	Moderate	High	Moderate	Low	Moderate	Moderate
Karhu et al., 2021 [22]	Longitudinal	2355	Low	Low	Low	Moderate	Low	Low
Kaya et al., 2020 [23]	Cross-sectional	266	Low	High	Moderate	Low	Low	Moderate
Coussa-Koniski et al., 2020 [24]	Longitudinal	663	Low	Low	Moderate	Moderate	Low	Low
Barreto et al., 2020 [25]	Cohort	102	Moderate	High	High	Low	Moderate	High
Almeneessier et al., 2020 [26]	Cross-sectional	32	High	High	High	Low	High	High
Hein et al., 2019 [27]	Cross-sectional	1272	Low	High	Moderate	Low	Moderate	Moderate

Discussion

The studies included in this review demonstrate a relationship between OSA and cardiovascular health, with significant evidence pointing toward an increased risk of hypertension, heart failure, and stroke. This discussion synthesizes key findings from the included studies, critically evaluating the methods and findings, and ultimately highlights the broader impact of OSA on cardiovascular outcomes.

Hypertension and Sleep Apnea

Hypertension is the most consistently reported cardiovascular comorbidity associated with OSA across multiple studies. Wilson et al. [6] reported that women with prenatal hypertension or pre-eclampsia had a 1.4 times higher prevalence of sleep-disordered breathing compared to controls, although no significant correlation was found between pre-eclampsia and OSA in this study. Despite this, the higher prevalence of OSA in hypertensive women indicates that sleep apnea may exacerbate or even trigger hypertensive conditions during pregnancy, a population that is often underrepresented in OSA studies.

Poka-Mayap et al. [20] reported a striking 64.1% prevalence of high-risk OSA in their cohort, with even higher rates in males (78.3%) compared to females (53%). Importantly, their study found a significant association between moderate-to-severe OSA and hypertension (OR = 3.24, 95% CI: 1.08-9.72, p = 0.036). This finding aligns with the broader literature that identifies sleep apnea as a significant risk factor for hypertension, particularly in males. The study’s large sample size (n = 359) and rigorous statistical analysis strengthen its conclusions, although it is limited by its cross-sectional design, which cannot establish causality.

Saraei et al. [19] provided further evidence by identifying a significant association between high BMI and multiple risk factors, such as hypertension (BP ≥ 140/90 mmHg), obesity (BMI > 35 kg/m²), and age > 50 years, in 59.9% of their 281 participants. This reinforces the well-established link between obesity, OSA, and hypertension. However, the study’s reliance on BMI as the sole measure of obesity may overlook other relevant factors such as body fat distribution, which has been shown to affect OSA severity and cardiovascular outcomes. Moreover, the cross-sectional design once again limits the ability to infer a causal relationship between OSA and hypertension.

Kaya et al. [23] focused on the impact of OSA on hypertensive patients and found that OSA was strongly associated with increased age, higher Epworth Sleepiness Scale (ESS) scores, and higher oxygen desaturation index (ODI). Their study showed that nocturnal oxygen desaturation was observed more frequently in hypertensive patients, with the ratio of nocturnal oxygen desaturation (NOD) to the total sleep time being significantly higher in hypertensive patients. This suggests that intermittent hypoxia during sleep is a key factor in the development of hypertension in OSA patients. However, while the study was robust, its findings could be strengthened by a longitudinal design to better establish the temporal relationship between OSA and hypertension development.

Impact of OSA on Stroke and Cardiovascular Events

Barreto et al. [25] highlighted the significant impact of OSA on stroke risk, reporting that one-third of their 102 participants experienced a wake-up stroke (WUS), with 50% of these patients having moderate-to-severe OSA. Type 2 diabetes emerged as an independent predictor of stroke in their cohort (OR = 2.76; CI 1.10-6.05), further emphasizing the complex interplay between OSA, diabetes, and stroke risk. The study's small sample size is a limitation, as it reduces the generalizability of the findings. Nevertheless, their focus on wake-up strokes, a relatively underexplored area, adds important insights into how sleep-disordered breathing may precipitate cerebrovascular events.

The study by Hein et al. [27] corroborates these findings by reporting that short sleep duration (<5 hours) and sleep fragmentation (index >18 per hour) significantly increased the risk of hypertension, which is a well-known precursor to stroke. Moreover, they found that prolonged use of short or intermediate half-life benzodiazepine receptor agonists (BZRAs) was linked to higher incidences of both hypertension and cardiovascular events. Their findings that 30.03% of individuals with sleep problems had hypertension underscore the strong link between sleep fragmentation and blood pressure dysregulation. This study’s large sample size (n = 1,272) strengthens the reliability of the results, although the cross-sectional nature of the study once again limits causal inference.

Role of Comorbidities and Demographics

Obesity and diabetes consistently emerged as key comorbidities that exacerbate the cardiovascular risks associated with OSA. Saraei et al. [19] found that 59.9% of their participants had a combination of multiple risk factors, including obesity and hypertension, which significantly increased the likelihood of sleep apnea. Coussa-Koniski et al. [24] added further evidence by reporting that 90% of OSA patients in their cohort had at least one comorbidity, with 60% having two to four comorbidities. The study also found that older age, male sex, and obesity were significantly correlated with OSA severity.

Interestingly, their findings revealed gender differences in OSA presentation. While men had higher OSA severity, women exhibited higher rates of comorbidities such as diabetes and hypertension. Women also tended to report more unusual symptoms of OSA, such as fatigue or insomnia, which may contribute to underdiagnosis. This highlights a key limitation in OSA research - many studies rely on traditional symptoms like snoring and observed apneas, which may not be as prevalent in women. As a result, women may receive delayed diagnoses, which can exacerbate their cardiovascular risk.

Oxygen Desaturation and Cardiovascular Impact

One of the most critical pathophysiological links between OSA and CVD is intermittent hypoxia, which triggers a cascade of physiological responses that increase cardiovascular risk. Karhu et al. [22] demonstrated that individuals with pre-existing cardiovascular conditions, such as hypertension and diabetes, faced a significantly higher risk of worsening intermittent hypoxemia. They found that elevation in ODI and desaturation duration was more pronounced in individuals with cardiovascular comorbidities, underscoring the impact of poor oxygenation on cardiovascular health.

Almeneessier et al. [26] also found that the ODI predicted changes in systolic blood pressure, highlighting the critical role of oxygenation in managing cardiovascular risks in OSA patients. The study’s focus on both REM and NREM sleep revealed no significant difference in desaturation events between these sleep stages, suggesting that oxygen desaturation, irrespective of sleep stage, plays a central role in the cardiovascular impact of OSA.

Broader Cardiovascular Impact of OSA

The cumulative evidence from these studies highlights the broad and multifaceted impact of OSA on cardiovascular health. The intermittent hypoxia and sleep fragmentation characteristic of OSA contribute to systemic inflammation, oxidative stress, and sympathetic nervous system activation, which collectively increase the risk of developing hypertension, stroke, and heart disease. The studies reviewed consistently found that individuals with OSA, particularly in its moderate-to-severe form, are at a significantly higher risk of adverse cardiovascular outcomes compared to those without the condition.

Importantly, these cardiovascular risks are magnified in populations with comorbid conditions such as obesity and diabetes, as demonstrated by Saraei et al. [16] and Coussa-Koniski et al. [24]. The high prevalence of OSA in these populations further emphasizes the need for early screening and intervention. Furthermore, the gender disparities in OSA presentation suggest that women may be underdiagnosed, leading to delayed treatment and potentially worse cardiovascular outcomes.

Studies reviewed provide evidence that OSA is a significant modifiable risk factor for CVDs, including hypertension, stroke, and heart failure. The repeated episodes of intermittent hypoxia, sleep fragmentation, and nocturnal oxygen desaturation associated with OSA contribute to endothelial dysfunction, systemic inflammation, and sympathetic nervous system overactivity, all of which drive CVD progression. The findings highlight the importance of early diagnosis and intervention, particularly in high-risk populations with comorbidities such as obesity and diabetes.

Future Recommendations

Future research should focus on longitudinal studies to establish clearer causal relationships and investigate the long-term impact of OSA treatment on cardiovascular outcomes. In addition, addressing gender disparities in diagnosis and increasing awareness of non-traditional OSA symptoms, particularly in women, will be crucial for improving patient outcomes.

## Conclusions

OSA is a major modifiable risk factor for CVDs, including hypertension and heart failure. Key mechanisms, such as intermittent hypoxia and systemic inflammation, link OSA to cardiovascular conditions. Despite its high prevalence in individuals with obesity and hypertension, current research faces limitations, including variable diagnostic criteria and underrepresentation of certain populations. More longitudinal studies with standardized protocols are needed. Public health efforts should focus on early screening and intervention to reduce the cardiovascular burden of OSA, especially in high-risk groups.
